# Severe and Regionally Widespread Increases in Tissue Urea in the Human Brain Represent a Novel Finding of Pathogenic Potential in Parkinson’s Disease Dementia

**DOI:** 10.3389/fnmol.2021.711396

**Published:** 2021-10-22

**Authors:** Melissa Scholefield, Stephanie J. Church, Jingshu Xu, Stefano Patassini, Federico Roncaroli, Nigel M. Hooper, Richard D. Unwin, Garth J. S. Cooper

**Affiliations:** ^1^Centre for Advanced Discovery & Experimental Therapeutics, Division of Cardiovascular Sciences, School of Medical Sciences, Faculty of Biology, Medicine and Health, The University of Manchester, Manchester Academic Health Science Centre, Manchester, United Kingdom; ^2^Faculty of Science, School of Biological Sciences, The University of Auckland, Auckland, New Zealand; ^3^Geoffrey Jefferson Brain Research Centre, Manchester Academic Health Science Centre, Manchester, United Kingdom; ^4^Division of Neuroscience and Experimental Psychology, School of Biological Sciences, Faculty of Brain and Mental Health, The University of Manchester, Manchester, United Kingdom; ^5^Division of Neuroscience & Experimental Psychology, School of Biological Sciences, Faculty of Biology, Medicine and Health, The University of Manchester, Manchester Academic Health Science Centre, Manchester, United Kingdom; ^6^Stoller Biomarker Discovery Centre & Division of Cancer Sciences, School of Medical Sciences, Faculty of Biology, Medicine and Health, The University of Manchester, Manchester, United Kingdom

**Keywords:** Parkinson’s disease dementia (PDD), Huntington’s disease (HD), Alzheimer’s disease (AD), urea-analysis, metabolomics (OMICS), mass spectrometry-LC-MS/MS

## Abstract

Widespread elevations in brain urea have, in recent years, been reported in certain types of age-related dementia, notably Alzheimer’s disease (AD) and Huntington’s disease (HD). Urea increases in these diseases are substantive, and approximate in magnitude to levels present in uraemic encephalopathy. In AD and HD, elevated urea levels are widespread, and not only in regions heavily affected by neurodegeneration. However, measurements of brain urea have not hitherto been reported in Parkinson’s disease dementia (PDD), a condition which shares neuropathological and symptomatic overlap with both AD and HD. Here we report measurements of tissue urea from nine neuropathologically confirmed regions of the brain in PDD and post-mortem delay (PMD)-matched controls, in regions including the cerebellum, motor cortex (MCX), sensory cortex, hippocampus (HP), substantia nigra (SN), middle temporal gyrus (MTG), medulla oblongata (MED), cingulate gyrus, and pons, by applying ultra-high-performance liquid chromatography-tandem mass spectrometry (UHPLC-MS/MS). Urea concentrations were found to be substantively elevated in all nine regions, with average increases of 3–4-fold. Urea concentrations were remarkably consistent across regions in both cases and controls, with no clear distinction between regions heavily affected or less severely affected by neuronal loss in PDD. These urea elevations mirror those found in uraemic encephalopathy, where equivalent levels are generally considered to be pathogenic, and those previously reported in AD and HD. Increased urea is a widespread metabolic perturbation in brain metabolism common to PDD, AD, and HD, at levels equal to those seen in uremic encephalopathy. This presents a novel pathogenic mechanism in PDD, which is shared with two other neurodegenerative diseases.

## Introduction

Parkinson’s disease (PD) is the second most common neurodegenerative condition after Alzheimer’s disease (AD; Erkkinen et al., 2018). PD is characterised mainly by motor dysfunction including bradykinesia, resting tremor, and rigidity. Up to 80% of patients with PD develop cognitive dysfunction during the course of their disease, usually within 20 years from the diagnosis, designated as Parkinson’s disease dementia (PDD; Hely et al., 2008). Neuropathologically, PD and PDD are characterised by extensive loss of dopaminergic neurons in the substantia nigra (SN), and accumulation and progressive spread of misfolded α-synuclein with formation of Lewy bodies and Lewy neuropil threads. Deposition of α-synuclein is thought to begin in the olfactory bulbs and lower brainstem and progress to the midbrain and eventually to the neocortex (Braak et al., 2003; Jellinger, 2019). The severity of α-synuclein deposition in the post-mortem brain is assessed using the Braak staging system.

A conclusive clinical diagnosis of PD/PDD can be challenging due to the heterogeneity of the condition, presence of comorbidities and overlap with other forms of movement disorders and dementia (Irwin et al., 2013; Walker et al., 2019). The hypothesis that different forms of dementia represent a spectrum with common pathogenetic mechanisms has been suggested (Perl et al., 1998; Jellinger and Korczyn, 2018). Studies that search for perturbations in different areas of the brain across different neurodegenerative conditions represent an approach to unveil potential common pathogenetic mechanisms. Previous metabolomics studies have investigated AD and HD and demonstrated widespread increases in brain-tissue urea in both these conditions despite their different clinical phenotypes and genetic alterations (Patassini et al., 2015, 2016; Xu et al., 2016; Handley et al., 2017).

Peripheral blood levels of urea have been reported in PD patients with discordant results including increase in plasma (Glaab et al., 2019), no change in serum (Hatano et al., 2016), decreases in the CSF (Trezzi et al., 2017), and decrease in whole blood concentrations (Troisi et al., 2019). Additionally, none of these studies of peripheral urea distinguished between PD with or without dementia. Conclusions on cerebral tissue levels of this metabolite cannot be inferred from these studies but to our knowledge, brain-tissue urea levels have not previously been reported in PDD.

## Materials and Methods

### Obtaining Tissue for Urea Quantification

For this study, tissues from nine brain regions from nine cases with definitely diagnosed PDD and nine controls were obtained from the University of Miami Brain Endowment Bank, Miami, FL, United States (part of the National Institute of Health NeuroBioBank network). Tissues were dissected from the following human-brain regions: middle temporal gyrus (MTG); motor cortex (MCX); primary visual cortex (PVC); hippocampus (HP); anterior cingulate gyrus (CG); cerebellum, at the level of the dentate nucleus (CB); SN; pons; and medulla oblongata (MED). All available patient metadata for cases and controls were obtained and are herein presented in Supplementary Tables A2, 3.

### Diagnosis and Severity of Parkinson’s Disease Dementia Cases

Cases and controls were diagnosed by the referring neuropathologists of the Miami Brain Endowment Bank. All cases were diagnosed to be of the alpha-synucleinopathy neocortical type, consistent with the clinical phenotype of PDD. Controls did not show any features of neurodegeneration or vascular pathology. The brains were assessed using either Braak staging (Braak et al., 2003) and/or McKeith’s staging criteria for Lewy body dementias (McKeith et al., 2005; see Supplementary Material A).

### Tissue Dissection

Brain samples were cut into sections of 50 mg (±5 mg) for urea quantification using a metal-free ceramic scalpel. Samples were stored in “Safe-Lok” microfuge Eppendorf tubes (Eppendorf AG; Hamburg, Germany) and stored at −80°C prior to extraction.

### Urea Quantification

Urea was quantified in brain sample by UHPLC-MS/MS. Brain samples were extracted in 50:50 (v/v) methanol:chloroform containing labelled urea internal standard [Urea-^15^N_2_ 98 atom% ^15^N, 99% (CP), 316830 Sigma-Aldrich, MO, United States]. Extraction blanks containing only the methanol:chloroform:internal standard solvent were prepared. Samples were lysed in a TissueLyser batch bead homogeniser (Qiagen, Manchester, United Kingdom). LC-MS grade water was then added to samples before separation of polar and non-polar phases by centrifugation at 2,400 × *g* for 15 min. The polar methanol phase was transferred to a fresh tube before being dried overnight in a Speedvac centrifugal concentrator (Savant Speedvac, Thermo Scientific, United Kingdom).

Once dried, 0.1% formic acid was added to samples. The resulting solution was transferred to 300-μl autosampler vials, with two blanks containing only 0.1% (v/v) formic acid also prepared. Standard solutions containing a labelled urea internal standard and corresponding unlabelled urea external standards (urea analytical standard, 56180 Supelco, PA, United States) were prepared, containing concentrations of 0–5,000 μM unlabelled urea in 0.1% v/v formic acid. Three QC samples were also prepared containing 10 μm labelled urea and 20, 200, and 2,000 μM unlabelled urea in 0.1% v/v formic acid.

Urea quantification was performed on a TSQ Vantage triple quadrupole mass spectrometer coupled with an Accela UHPLC system (Thermo Fisher Scientific, MA, United States). Separation was carried out on a Hypersil Gold AQ column with a diameter of 2.1 mm, length of 100 mm, and particle size of 1.9 μm (Thermo Fisher Scientific) maintained at 25°C with a 0.5 μm pre-column filter (Thermo Fisher Scientific). Gradient elution was performed using 0.1% formic acid in water (A) and 0.1% formic acid in acetonitrile (B) at 300 μl/min. Urea and labelled urea internal standard were detected using electrospray ionisation in positive ionisation mode.

### Ultra-High-Performance Liquid Chromatography-Tandem Mass Spectrometry Data Analysis

Ultra-high-performance liquid chromatography-tandem mass spectrometry data were analysed using LCQuan software (Thermo Fisher Scientific, MA, United States). Chromatographic peaks were identified based on expected retention time (RT), and compared against labelled urea internal standard peak RTs for each individual QC/standard/sample. Each peak was manually checked for correct identification.

Quantification of urea in samples was performed using the ratio of urea peak area to internal standard peak area and comparison to the standard curve. These concentrations were corrected for sample wet-weight and analysed in GraphPad Prism v8.1.2. (Prism; La Jolla, CA, United States). A non-parametric Mann-Whitney-*U* test was used to determine the significance of case-control differences due to the relatively small sample sizes.

The minimum sample size required to confidently determine case-control differences at a significance level of *p* < 0.05 and *p* < 0.01 was calculated using the sample size calculator from SPH Analytics, Alpharetta, United States.^1^

## Results and Discussion

### Cohort Characterisation

Metadata were obtained for all cases and controls, including sex, age, race, ethnicity, post-mortem delay (PMD), brain weight, comorbidities, and cause of death (see Supplementary Material A for individual data). There were no significant case-control differences in sex, age at death, PMD or brain weight (Supplementary Table A1). All samples had a PMD of 26 h or less, with an average of 19.8 h in controls and 14.6 h in cases.

Due to a lack of available SN tissue for two of the cohort samples, two alternate SN samples were obtained from different donors. These samples were age- and sex-matched, but the cases had a lower PMD than controls (∼6 h, *p* = 0.03; Supplementary Table A1). The impact of this is discussed in the section on brain tissue urea findings below.

### Brain Tissue Urea Findings in Parkinson’s Disease Dementia

**FIGURE 1:**
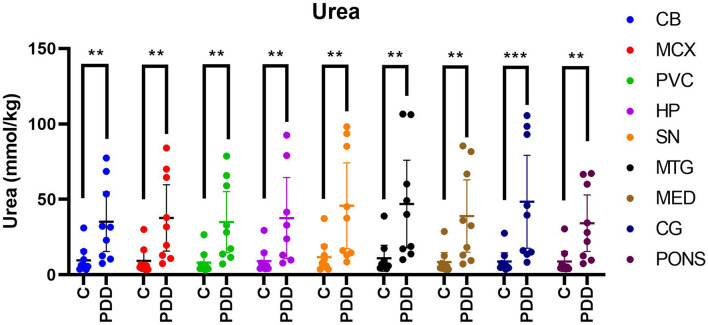
Urea concentrations in PDD cases and matched controls. Mean tissue urea concentrations ± 95% confidence intervals expressed in mmol/kg. Case-control differences were determined by applying the Mann-Whitney-*U* test. **p < 0.01 and ***p < 0.001. C, control; PDD, Parkinson’s disease dementia case.

Urea levels were increased in PDD cases compared to controls in every region analysed (Table 1 and Figure 1). There was an average 5.5-fold increase in tissue-urea concentrations in the CG of cases compared with controls, and an average ∼3.5- to 4.5-fold increase in cases in every other region.

**TABLE 1:** Tissue urea concentrations in PDD cases and controls.

Region	Controls (*n* = 9) (mmol/kg)	PDD cases (*n* = 9) (mmol/kg)	Fold-change	*P*-Value
CB	9.6 (2.7–16.4)	35.2 (15.4–55.0)	**3.7**	**0.003**
MCX	9.2 (2.5–16.0)	37.7 (15.7–59.7)	**4.1**	**0.002**
PVC	8.1 (2.3–13.9)	34.9 (14.6–55.2)	**4.3**	**0.001**
HP	9.0 (2.6–15.4)	37.6 (10.7–64.4)	**4.2**	**0.004**
SN	11.6 (3.4–19.9)	45.8 (17.3–74.2)	**3.9**	**0.006**
MTG	11.0 (2.3–19.6)	46.9 (17.9–76.0)	**4.3**	**0.002**
MED	8.5 (2.3–14.7)	38.9 (14.9–62.9)	**4.6**	**0.002**
CG	8.8 (3.0–14.6)	48.4 (17.6–79.1)	**5.5**	**0.0008**
PONS	8.8 (2.2–15.5)	34.2 (15.4–53.0)	**3.9**	**0.003**

*Mean tissue-urea concentrations ± 95% confidence intervals expressed in mmol/kg. Significance of case-control differences was determined using the Mann-Whitney-*U* test wherein *P* < 0.05 has been considered significant. Fold-changes are cases compared to controls.*

*Bolded values show significant case-control differences.*

Inter-regional concentrations of urea were highly consistent in both controls and cases, with no significant differences between any two regions. Control tissue-urea concentrations averaged ∼9.4 mmol/kg and case concentrations 40.0 mmol/kg, showing a substantial, ∼4-fold increase in case-control urea levels across the brain overall.

Neither of the substituted SN samples showed significant differences in urea concentrations compared with other cases in the cohort (see Supplementary Material B for individual values). Case-control differences in SN-urea levels remained significant with exclusion of substituted SN controls C10 and C11.

As sample sizes in this study were small, a study measuring the statistical power of the urea values was conducted. This found a statistical power of over 80% in the CB, MCX, PVC, MED, CG, and PONS, over 70% in the SN and MTG, and a lower value of 68.7% in the HP for *p* < 0.05. This indicates good statistical power in most regions, but highlights the necessity of larger sample sizes for more robust measurements. The same test showed a required sample size of 3 for confident identification of case-control differences at *p* < 0.05 for all regions except the HP, which showed a required sample size of 4.

### Comparisons With Brain Urea Concentrations in Alzheimer’s Disease and in Huntington’s Disease

**FIGURE 2:**
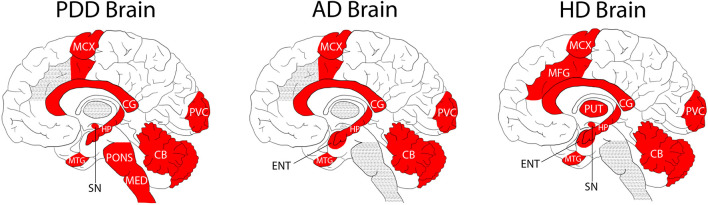
Regional Distribution of Measured Tissue Urea Increases in the PDD, AD, and HD Brain. Areas shaded in red denote significant increases in urea compared to intra-cohort controls. Areas shaded in grey were not investigated in the illustrated studies.

Our group has previously reported severe and regionally widespread increases in brain-tissue urea concentrations in cases with AD dementia (Xu et al., 2016), and with HD dementia (Patassini et al., 2015, 2016). In these studies, AD and HD brain-tissue urea values were reported from multiple regions compared between cases and matched controls [Table 2 and Figure 2; refs (Patassini et al., 2015, 2016; Xu et al., 2016)].

**TABLE 2:** Urea fold-changes in PDD, AD, and HD.

Region	Fold-change in PDD (this study)	Fold-changes in AD (Xu et al., 2016)	Fold-changes in HD (Patassini et al., 2015)
CB	3.7	4.9	3.6
MCX	4.1	5.0	3.4
PVC	4.3	4.9	3.4
HP	4.2	6.5	3.6
SN	3.9	–	3.5
MTG	4.3	4.7	3.4
MED	4.6	–	–
CG	5.5	5.3	3.5
PONS	3.9	–	*–*
PUT	*–*	–	3.7
GP	*–*	–	3.6
MFG	*–*	–	3.0
ENT	*–*	5.6	2.8
Overall	4.3 (3.9–4.6)	5.3 (4.8–5.8)	3.5 (3.2–3.6)

*Comparisons of case-control fold-changes in human brain urea levels between PDD, AD, and HD. Case-control differences were significant for every region in every disease. Overall shows mean overall fold-change with ± 95% confidence intervals.*

Although there are some differences in the methodologies used in the two former studies compared with the current one (such as previous use of gas chromatography-mass spectrometry, and some differences in the regions studied; Figure 2), case-control tissue-urea fold-changes can be compared between PDD, HD, and AD (Table 2) across several brain regions.

Cases and controls in the HD study were slightly younger than those in the current PDD cohort, at an average of around 3 years for controls and 8 years for cases (Patassini et al., 2015, 2016). Moreover, PMD was significantly lower than in the current PDD cohort, at an average of ∼11 h in both cases and controls. Cause of death in the HD cohort was most commonly bronchopneumonia in cases and heart failure in controls, but data on comorbidities and other vascular pathology were unavailable. Brain tissue-urea concentrations showed a similar average fold-increase to that measured in PDD of 3.5 in HD cases compared to controls (Table 2 and Figure 2). As in PDD, the increase was observed in the SN, CB, HP, MTG, CG, SCX, and MCX, as well as in other regions not included in the current PDD study, such as the putamen, globus pallidus, middle frontal gyrus, and entorhinal cortex.

The AD cases whose brain-tissue urea levels we measured were similar in age to the current PDD cohort, with an average of ∼70 years in both cases and controls (Xu et al., 2016). PMD was also significantly shorter in this cohort, at an average of 9 h in controls and 7 h in cases. Causes of death were varied in both cases and controls, most commonly being due to heart or lung complications. Comorbidity and vascular pathology metadata were not available. Although absolute tissue-urea concentrations cannot be directly compared to the previous studies in AD brains, there was a higher average case-control fold-change in AD than that observed here in PDD, the average being ∼5.3-fold, but with increases as high as 6.5-fold in the HP (Xu et al., 2016; Table 2). Tissue-urea elevations in this AD cohort were observed in the HP, MTG, SCX, MCX, CG, and CB, as well as in the entorhinal cortex (which was not included in the current study). The AD cohort was similar in age to the current PDD cohort.

Comparisons of case-control fold-changes in human brain urea levels between PDD, AD, and HD. Case-control differences were significant for every region in every disease. Overall shows mean overall fold-change with ± 95% confidence intervals.

Additional studies have also confirmed urea increases in AD (Gueli and Taibi, 2013) and HD (Handley et al., 2017) brains, although one study reported decreases in the HD striatum and no change in the HD frontal lobe (Graham et al., 2016). Such discrepancies may be contributed to by differences in methods used or between cohorts, such as differences in PMD. However, we have previously observed that PMD does not affect urea levels for up to 72 h in rat brains (Scholefield et al., 2020), although this may not necessarily also be the case in human brains.

Together, the observations in PDD, AD, and HD suggest a shared pathogenic mechanism in these three diseases, despite apparent differences in causative processes and symptomology. There appear to be regional differences between diseases with respect to tissue-urea elevations. For example, AD showed the highest increases in the severely affected HP (6.5-fold), whereas in HD the putamen showed slightly higher-than-average elevations (3.7-fold). These regional differences may contribute to differences in severity and pathogenesis in the different conditions. Interestingly, the CG showed the highest urea increase in PDD (5.5-fold); the anterior CG is involved in emotive states and emotionally-coded memories. All other studied regions of the PDD brain showed urea increments of around 3.5- to 4.5-fold. However, there were no statistically significant differences between different PDD-control regions or between different PDD-case regions. Greater power (by increasing the sample size), would probably be required to determine whether the fold-elevation observed in the CG is significantly greater than that of other regions of the PDD brain. If so, it is possible that the prominence of both motor and cognitive dysfunction in PDD correlates with similar tissue urea elevations in areas involved in both cognition and motor control processes–a future investigation into regional tissue urea dysregulation in PD brains without dementia could help elucidate this and would probably be a logical next step.

Urea’s main metabolic function is to provide a route for the excretion of nitrogen-containing moieties derived from toxic nitrogen-containing compounds. In the urea cycle, ammonia is converted into urea, which is then excreted from the body *via* the urine. Urea is mainly produced in the liver and is carried to other parts of the body *via* the blood stream. However, it is slow to cross the blood-brain barrier (BBB; Sterns et al., 2015), leading to the question as to whether cerebral urea does in fact enter the brain through the BBB, or whether it is also produced in the brain. There is little evidence for the presence of intracerebral urea cycle activity, although there have been limited observations of partial activity in some studies. For example, some studies have identified increased expression of arginase I (ARG1) mRNA in AD brains and the brains of the APP^sw^ mouse model of AD (Colton et al., 2006), as well as increased arginase activity in human AD brains–accompanied by concurrent decreases in ornithine (Liu et al., 2014). Arginase is the final enzyme of the urea cycle, responsible for conversion of arginine to urea–as such, increased arginase activity could result in increased urea levels. A proteomics study conducted by our own lab on several regions of the AD brain observed arginase II in the CB, but did not observe any case–control changes in the enzyme; ARGI was not identified in any region (Xu et al., 2019). Triple transgenic AD mouse models have also exhibited reduced amyloid-β deposits, microgliosis, and spatial memory deficits with administration of arginase inhibitor L-norvaline (Polis et al., 2018). Administration of an arginase-1 inhibitor has also been found to be protective against midbrain dopaminergic neuronal loss induced in rat brain slice cultures by arginase-1 promoter macelignan (Kiyofuji et al., 2015). A network analysis has also found arginine pathways be enriched in both PD and AD, as well as in amyotrophic lateral sclerosis (Kori et al., 2016), possibly resulting in downstream upregulation of nitric oxide synthesis, leading to hypoxia, which has been implicated in both PD and AD (Virarkar et al., 2013). Together, these results suggest a role for arginase in AD and PD pathology.

However, arginase is only one component of the urea cycle and is not sufficient for complete urea cycle activity; another urea cycle component–ornithine transcarbamylase (OTC)–the enzyme responsible for the conversion of ornithine to citrulline in the urea cycle, was initially reported to be expressed only in AD brains (Bensemain et al., 2009), but has also been observed in healthy control brains in limited amounts (Bernstein et al., 2015, 2017). However, studies attempting to investigate the levels of other urea cycle enzymes in HD sheep model brains for example were unable to identify either OTC or carbamoyl phosphate synthetase I in the striatum (Handley et al., 2017). Additionally, a more recent large-scale proteomic study of six regions of human AD and control brains was unable to identify the presence of either of these urea cycle enzymes at the protein level (Xu et al., 2019). As such, it is possible that upregulation of OTC in AD brains leads to urea cycle activity in the brain that would otherwise not be present, characterised by increased urea and arginase activity and decreased ornithine, but the lack of other urea cycle components makes definite conclusions difficult to draw at this stage. It is not yet known whether increases in OTC are also present in the PDD brain.

Some other urea cycle components such as adenosine (McFarland et al., 2013; Xu et al., 2016; Alonso-Andres et al., 2018), citrulline (Liu et al., 2014), and ornithine (Gueli and Taibi, 2013; Liu et al., 2014) have been reported in the AD brain, as well as the HD brain (Graham et al., 2016; Patassini et al., 2016)—however, as these metabolites are also involved in other metabolic pathways, this does not necessarily indicate urea cycle activity. For example, adenosine is a core component of several widespread co-enzymes such as adenosine triphosphate (ATP), diphosphate (ADP), and monophosphate (AMP) which are crucial to a wide variety of metabolic pathways including the ETC (Nolfi-Donegan et al., 2020), TCA cycle (Madeira, 2018), and purine metabolism (Ichida et al., 2009). As such, it seems likely that cerebral urea may be formed by an alternative process, either by itself or in addition to potential urea cycle activity in the brain.

Several urea cycle intermediates have also been reported to be dysregulated in PD serum, plasma, and CSF. Arginine has been reported by some investigations to be decreased in PD serum (Mally et al., 1997) and CSF (Molina et al., 1997), although other reports have observed no changes in either the serum (Hatano et al., 2016), CSF (Mally et al., 1997; Engelborghs et al., 2003), or plasma (Molina et al., 1997). There is one report of increased citrulline in PD serum (Han et al., 2017), although another investigation reported no change (Hatano et al., 2016), and further reports indicated no change in the CSF (Molina et al., 1997; Engelborghs et al., 2003) or plasma (Iwasaki et al., 1992; Molina et al., 1997). Increases in PD serum ornithine have been reported (Hatano et al., 2016), with varying reports in the CSF of decrease (Molina et al., 1997), increase (Wuolikainen et al., 2016), or no change (Engelborghs et al., 2003). Moreover, no changes have been reported by several groups in studies of plasma ornithine in PD (Iwasaki et al., 1992; Molina et al., 1997; Wuolikainen et al., 2016). None of these urea cycle components have been reported on in the PD/PDD brain itself, and the investigations of peripheral levels in the plasma, CSF, and serum have reported inconsistent results. As such, this entire pathway, and the possibility of partial or whole urea cycle activity in the brain, presents a novel area for future investigation of PDD.

Protein dysregulation and neuronal death may lead to greater protein breakdown, and so to increased urea production. Disruptions to the BBB, as observed in PDD (Sweeney et al., 2018), may result in defective urea clearance from the brain or increased entry of urea from the bloodstream *via* urea transporters. Urea transporters are responsible for regulating movement of urea by facilitating urea diffusion, and are expressed in astrocytes and the BBB as well as outside the brain. Urea transporter B has been shown to be upregulated in the HD CB, which may reflect attempts to clear elevated urea levels in the HD brain (Handley et al., 2017). Urea transporters have not yet been investigated in PD/PDD, but may show similar perturbations to those reported in HD.

Urea accumulation caused by kidney failure is known to be toxic to the brain, leading to a condition called uraemic encephalopathy (Seifter and Samuels, 2011). High urea levels lead to synaptic loss and inhibition of long-term potentiation *via* carbamylation of mTOR in a chronic kidney disease mouse model (Wang H. et al., 2019). Carbamylation is a post-translational modification involving the addition of isocyanate, usually derived from urea, to protein-bound amino-acid residues, causing alterations in the structure and function of the affected proteins. Increased carbamylation has been observed in aging humans (Gorisse et al., 2016), and as a result of chronic kidney disease (Long et al., 2018) as well as in AD with cerebrovascular disease (Gallart-Palau et al., 2017). It has been shown that tau, which is aggregated in AD and also to a lesser degree in PDD, can be carbamylated, resulting in increased amyloid formation and tau accumulation (Guru KrishnaKumar et al., 2018). Whether α-synuclein may also be carbamylated is unknown, but it does contain several potentially susceptible amino-acid residues, which might serve as target sites for carbamylation.

High urea levels in chronic kidney disease and renal failure have also been linked to increased oxidative stress (Vaziri, 2004). Oxidative stress is a well-recognised feature of PD, AD, and HD (Yan et al., 2013; Graham et al., 2016) and is linked to other pathogenic mechanisms including mitochondrial dysfunction and proteinopathy (Ganguly et al., 2017), dysregulated glucose metabolism (Butterfield and Halliwell, 2019), insulin resistance and inflammation (Verdile et al., 2015), and α-synuclein accumulation, oligomerisation, and phosphorylation (Scudamore and Ciossek, 2018; Wang R. et al., 2019). As such, it is possible that elevated urea levels in PDD could contribute to one or more of these known pathogenic mechanisms.

Weaknesses of the current study include small sample sizes of a maximum of 9 v 9 in all three diseases. However, analyses of statistical power show good values, at >80% in most PDD brain regions. Non-parametric tests were also employed due to the small sample size in order to avoid type I errors during statistical analysis. Despite this, larger sample sizes would lead to more robust findings. There are also some regions of the PDD brain that may have been of interest for comparison with other dementias, such as the putamen and caudate nucleus, which are both heavily affected in both PDD and HD. However, this study was limited by the number of available regions for the same donors (which allow for more direct intraregional comparisons of urea concentrations), and also due to our aim of including not only heavily affected regions, but also moderately affected and relatively spared regions. As such, future investigations should try to include such areas, which to our knowledge have yet to be investigated in terms of urea concentrations in PDD. An additional weakness in this study is a lack of additional metadata, such as the duration of Parkinsonism and dementia symptoms in PDD cases, or data on renal functions in donors. Unfortunately, this data was not available for the tissues obtained in this study; although no cases or controls were reported as having renal issues involved in their cause of death, it is unknown whether they had any kidney-related comorbidities before death.

The strengths of this study are the use of highly precise quantitative UHPLC-MS/MS methods, performed in such a way as to make the measured values directly comparable to brain tissues from previous analyses of other dementia diseases. The cohort used for this study was also very well-matched, with only a small difference in PMD between SN cases and controls. Data was also obtained on donor cause of death and neuropathology–as provided by referring neuropathologists at the brain bank–which is not always available in studies such as these. Statistical tests of study power showed values of >80% in most regions, suggesting good statistical power at *p* > 0.05. Although larger sample sizes are required for further studies, this suggests that a good amount of confidence can be had in the results reported here.

## Conclusion

In conclusion, this investigation shows widespread urea accumulation throughout the PDD brain, similar to that previously observed in AD and HD, with concentrations comparable to those seen in uremic encephalopathy. This suggests a novel shared pathogenic insult across multiple neurodegenerative conditions and may indicate a common mechanism in the development of cognitive impairment.

## Data Availability Statement

The original contributions presented in the study are included in the article/Supplementary Material, further inquiries can be directed to the corresponding author/s.

## Ethics Statement

The studies involving human participants were reviewed and approved by Manchester REC (09/H0906/52C5). The patients/participants provided their written informed consent to participate in this study.

## Author Contributions

MS designed and performed research, analysed and interpreted data, and wrote the first draft and subsequent drafts of the manuscript. SC performed research, analysed data, and read and revised the manuscript. FR, NH, and RU read and revised the manuscript. GC conceived, designed and supervised research, analysed and interpreted data, wrote the manuscript, and bears overall responsibility for the integrity of the study and of the manuscript. JX performed research on previous Auckland AD cohort. SP performed research on previous HD cohort. All authors contributed to the article and approved the submitted version.

## Conflict of Interest

The authors declare that the research was conducted in the absence of any commercial or financial relationships that could be construed as a potential conflict of interest.

## Publisher’s Note

All claims expressed in this article are solely those of the authors and do not necessarily represent those of their affiliated organizations, or those of the publisher, the editors and the reviewers. Any product that may be evaluated in this article, or claim that may be made by its manufacturer, is not guaranteed or endorsed by the publisher.

## Footnotes

1
https://www.sphanalytics.com/sample-size-calculator-using-average-values/
