# Supplementary material for: Severe and Regionally Widespread Increases in Tissue Urea in the Human Brain Represent a Novel Finding of Pathogenic Potential in Parkinson’s Disease Dementia
Source: Front Mol Neurosci. 2021 Oct 22;14:711396. doi: 10.3389/fnmol.2021.711396 (PMC8571017; doi:10.3389/fnmol.2021.711396)
Supplement: Supplementary file 1 [file Table_1.DOCX]

Supplementary Material A

**Contents**

[Supplementary Table 1. Cohort Characteristics 2](#_Toc72235141)

[Supplementary Table 2: Characteristics of individuals in the PDD cohort 3](#_Toc72235142)

[Supplementary Table 3: Characteristics of controls in the SN cohort 6](#_Toc72235143)

[Supplementary Table 4: Statistical Power of Urea Analyses 7](#_Toc72235144)

[Supplementary Table 5: Urea Concentrations in PDD and HD 8](#_Toc72235145)

## Supplementary Table 1. Cohort Characteristics

|  | Gender  (% male) | Age at death (years) | PMD  (hours) | Brain Weight (g) |
| --- | --- | --- | --- | --- |
| Controls (n = 9) | 44 | 70 (61 – 79) | 19.8 (12.5 – 26.0) | 1285 (1135 – 1371) |
| SN Controls (n = 9) | 44 | 70 (62 – 79) | 20.6 (10.8 – 26.0) | 1335 (1135 – 1550)† |
| Cases  (n = 9) | 66 | 73 (61 – 81) | 14.6 (4.3 – 21.9) | 1291 (1187 – 1520) |

Mean (range) age, PMD, and brain weight. * p < 0.05 between cases and controls as determined by Mann-Whitney-U Test; all other differences were not significant. †Brain weight not available for one control.

## Supplementary Table 2: Characteristics of individuals in the PDD cohort

| Code | Sex | Age at death | Clinical diagnosis | PD Neuropathology | Post-Mortem Delay (hours) | Whole-brain weight (g) | Cause of Death |
| --- | --- | --- | --- | --- | --- | --- | --- |
| C1 | Male | 61 | No dementia present | None observed | 12.5 | 1182 | Respiratory failure; heart failure; coronary artery disease |
| C2 | Male | 71 | No dementia present | None observed | 23.9 | 1371 | Acute myocardial infarction; severe coronary artery disease; pulmonary oedema; diabetes mellitus; cardiopulmonary arrest |
| C3 | Male | 74 | No dementia present | None observed | 25.5 | 1300 | Aortic dissection |
| C4 | Male | 70 | No dementia present | None observed | 12.7 | 1350 | Atherosclerotic and hypertensive heart disease |
| C5 | Female | 68 | No dementia present | None observed | 19.1 | 1270 | Acute myocardial infarction; coronary artery disease |
| C6 | Female | 65 | No dementia present | None observed | 19.4 | 1372 | Hypertensive arteriosclerotic cardiovascular disease; morbid obesity; respiratory arrest; suspected embolus |
| C7 | Female | 77 | No dementia present | None observed | 21.4 | 1135 | Pending death certificate |
| C8 | Female | 79 | No dementia present | None observed | 17.8 | 1300 | Acute myocardial infarction; coronary artery disease; atrial fibrillation; COPD |
| C9 | Female | 67 | No dementia present | None observed | 25.9 | 1382 | Pending death certificate |
| PDD1 | Male | 61 | Parkinson’s disease dementia | Limbic (transitional) | 13.8 | 1188 | Cardiopulmonary arrest; probable acute myocardial infarction; Parkinson’s disease |
| PDD2 | Male | 79 | Parkinson’s disease dementia | Limbic (transitional) | 16.2 | 1250 | End stage Parkinson’s disease |
| PDD3 | Male | 71 | Parkinson’s disease dementia | Brainstem predominant; Braak stage III-IV | 16.2 | 1262 | Pending death certificate |
| PDD4 | Male | 78 | Parkinson’s disease dementia | Diffuse neocortical; Braak stage VI | 20.4 | 1520 | Respiratory failure; aspiration pneumonia; dysphagia; Parkinson’s disease |
| PDD5 | Male | 70 | Parkinson’s disease dementia | Diffuse neocortical | 4.3 | 1218 | Aspiration pneumonia; Parkinson’s disease |
| PDD6 | Female | 69 | Parkinson’s disease dementia | Braak stage IV-V | 17.5 | 1187 | Pending death certificate |
| PDD7 | Female | 81 | Parkinson’s disease dementia | Diffuse neocortical | 7.0 | 1415 | End stage Parkinson’s disease |
| PDD8 | Female | 79 | Parkinson’s disease dementia | Limbic (transitional) | 21.9 | 1200 | Respiratory failure; Parkinson’s disease |
| PDD9 | Female | 67 | Parkinson’s disease dementia | Diffuse neocortical | 14.5 | Not available | Lewy body disease; Parkinson's disease |

## Supplementary Table 3: Characteristics of controls in the SN cohort

| Code | Sex | Age at death | Clinical diagnosis | PD Neuropathology | Post-Mortem Delay (hours) | Whole-brain weight (g) | Cause of Death |
| --- | --- | --- | --- | --- | --- | --- | --- |
| C2 | Male | 71 | No dementia present | None observed | 23.9 | 1371 | Acute myocardial infarction; severe coronary artery disease; pulmonary oedema; diabetes mellitus; cardiopulmonary arrest |
| C3 | Male | 74 | No dementia present | None observed | *25.5* | 1300 | Aortic dissection |
| C5 | Female | 68 | No dementia present | None observed | 19.1 | 1270 | Acute myocardial infarction; coronary artery disease |
| C6 | Female | 65 | No dementia present | None observed | *19.4* | 1372 | Hypertensive arteriosclerotic cardiovascular disease; morbid obesity; respiratory arrest; suspected embolus |
| C7 | Female | 77 | No dementia present | None observed | 21.4 | 1135 | Pending death certificate |
| C8 | Female | 79 | No dementia present | None observed | 17.8 | 1300 | Acute myocardial infarction; coronary artery disease; atrial fibrillation; COPD |
| C9 | Female | 67 | No dementia present | None observed | 25.9 | 1382 | Pending death certificate |
| C10 | Male | 68 | No dementia present | None observed | 10.8 | 1550 | Pending death certificate |
| C11 | Male | 62 | No dementia present | None observed | 21.3 | Not available | Diabetes; triple vessel disease of the heart; hyperlipidaemia |

## Supplementary Table 4: Statistical Power of Urea Analyses

| Element | Statistical Power (p < 0.05) | Statistical Power (p < 0.01) | Sample Size Required (p < 0.05) | Sample Size Required (p < 0.01) |
| --- | --- | --- | --- | --- |
| CB | **80.6** | 59.8 | **3** | **6** |
| MCX | **81.5** | 61.1 | **3** | **6** |
| PVC | **83.4** | 63.8 | **3** | **6** |
| HP | 68.7 | 44.9 | **4** | **7** |
| SN | 75.9 | 53.4 | **3** | **7** |
| MTG | 78.0 | 56.2 | **3** | **7** |
| MED | **80.8** | 60.0 | **3** | **6** |
| CG | **83.1** | 63.4 | **3** | **6** |
| PONS | **83.4** | 63.9 | **3** | **6** |

Bold values indicate statistical power of > 80% or sample size of < 10

## Supplementary Table 5: Urea Concentrations in PDD and HD

| Region | PDD Controls (n = 9)  (mmol/kg) | HD Controls (n = 9)  (mmol/kg) | PDD Cases (n = 9)  (mmol/kg | HD Cases (n = 9)  (mmol/kg) |
| --- | --- | --- | --- | --- |
| CB | 9.6 (2.7 - 16.4) | 7.3 (6.2 – 8.3) | 35.2 (15.4 - 55.0) | 26.2 (15.3 – 37.1) |
| MCX | 9.2 (2.5 - 16.0) | 7.8 (6.5 – 9.1) | 37.7 (15.7 - 59.7) | 26.4 (14.5 – 38.3) |
| PVC | 8.1 (2.3 - 13.9) | 7.4 (6.1 – 8.6) | 34.9 (14.6 - 55.2) | 24.6 (14.4 – 34.8) |
| HP | 9.0 (2.6 - 15.4) | 6.7 (5.6 – 7.8) | 37.6 (10.7 - 64.4) | 24.0 (12.9 – 35.1) |
| MTG | 11.0 (2.3 - 19.6) | 8.6 (7.3 – 9.8) | 46.9 (17.9 - 76.0) | 28.8 (17.6 – 40.0) |
| CG | 8.8 (3.0 - 14.6) | 7.1 (6.0 – 8.2) | 48.4 (17.6 - 79.1) | 24.8 (13.9 – 35.8) |
| SN | 11.6 (3.4 - 19.9) | 6.5 (5.3 – 7.7) | 45.8 (17.3 - 74.2) | 23.0 (13.3 – 32.7) |

HD data acquired from Patassini et al, 2015
